# Diagnosis of Langerhans cell histiocytosis on cytological examination of cerebrospinal fluid: Report of the first case

**DOI:** 10.1002/dc.25040

**Published:** 2022-08-11

**Authors:** Francesco Tommasino, Chiara Cardamone, Vincenzo Tortora, Francesco Sabbatino, Chiara Di Sarno, Alessandro Caputo

**Affiliations:** ^1^ Department of Medicine and Surgery University of Salerno Salerno Italy; ^2^ Department of Medicine University of Naples “Federico II” Naples Italy; ^3^ Department of Precision Medicine University of Naples “Luigi Vanvitelli” Naples Italy

**Keywords:** cerebrospinal fluid, cytology, langerhans cell histiocytosis

## Abstract

Langerhans cell histiocytosis (LCH) is a disease of unknown etiology characterized by a proliferation of histiocytic cells resembling dendritic Langerhans cells. LCH can be unifocal or multifocal, with one‐ or many‐organ involvement. The serous fluids are rarely involved. Cytological diagnosis of LCH is possible and relies on recognition of the typical cytomorphological features and subsequent immunocytochemical confirmation. Given the possibility of multisystem involvement, after diagnosing LCH it is necessary to carry out staging exams such as a bone survey, abdominal ultrasound, complete blood count, screening for diabetes insipidus and pulmonary function tests. We present the first case of LCH where the diagnosis was reached on cytological material from the cerebrospinal fluid. To the best of our knowledge, this is the first such case reported in the international literature to date. The morphological and immunocytochemical characteristics of our case are described, and the relevant literature is reviewed

## 1 INTRODUCTION

Histiocytoses are a heterogeneous group of diseases characterized by proliferation of mononuclear phagocytes. Langerhans cell histiocytosis (LCH) consists in a clonal proliferation of antigen‐presenting dendritic cells with ultrastructural and phenotypic characteristics of Langerans cells.^1^

LCH is more common in children than adults, with a reported incidence ranging from 2.6 to 8.9 cases per million children (<15 years) per year and 0.07 cases per million adults per year.^1^ LCH has a broad clinical spectrum since it can affect a single site or multiple organ systems, simultaneously or sequentially. The most typical site of involvement is bone: about 80% of patients with LCH present with lytic bone lesions, and typical locations include the skull, jaw, and femur. Other organs commonly affected are skin, most commonly in children; lungs, with cysts and nodules; central nervous system with potential involvement of pituitary gland and development of diabetes insipidus.^2^ The serous cavities can rarely be involved by LCH.^3^

Diagnosis requires histological analysis, which shows the characteristic microscopic features of LCH: numerous dyscohesive Langerhans cells deeply intermixed with an inflammatory population comprising lymphocytes, neutrophils, and eosinophils. The Langerhans cells have single (rarely multiple), round or oval nuclei with deep convolutions and grooves. Atypical features include giant nucleoli, pleomorphism, elongated cells with cytoplasmic tails, and intracytoplasmic vacuoles.^4^ The Langerhans cells will be immunohistochemically positive for langerin (CD207) and CD1a—the two most specific markers—as well as S100, CD4, CD68, and lysozyme. Birbeck granules, a typical ultrastructural feature of Langerhans cells, can be detected with electron microscopy.

Given the possibility of multisystem involvement, after diagnosing LCH it is necessary to carry out staging exams such as a bone survey, abdominal ultrasound, complete blood count, screening for diabetes insipidus and pulmonary function tests.^5^

To date, only one case of LCH involving the cerebrospinal fluid (CSF) has been reported. In this case, the diagnosis had previously been reached on lymph node biopsy and a CSF localization was then confirmed on cytological material.^6^ We report instead the first case in which the diagnosis was initially suspected on cytological examination of the CSF and then confirmed histologically.

## 2 CASE REPORT

**FIGURE 1:**
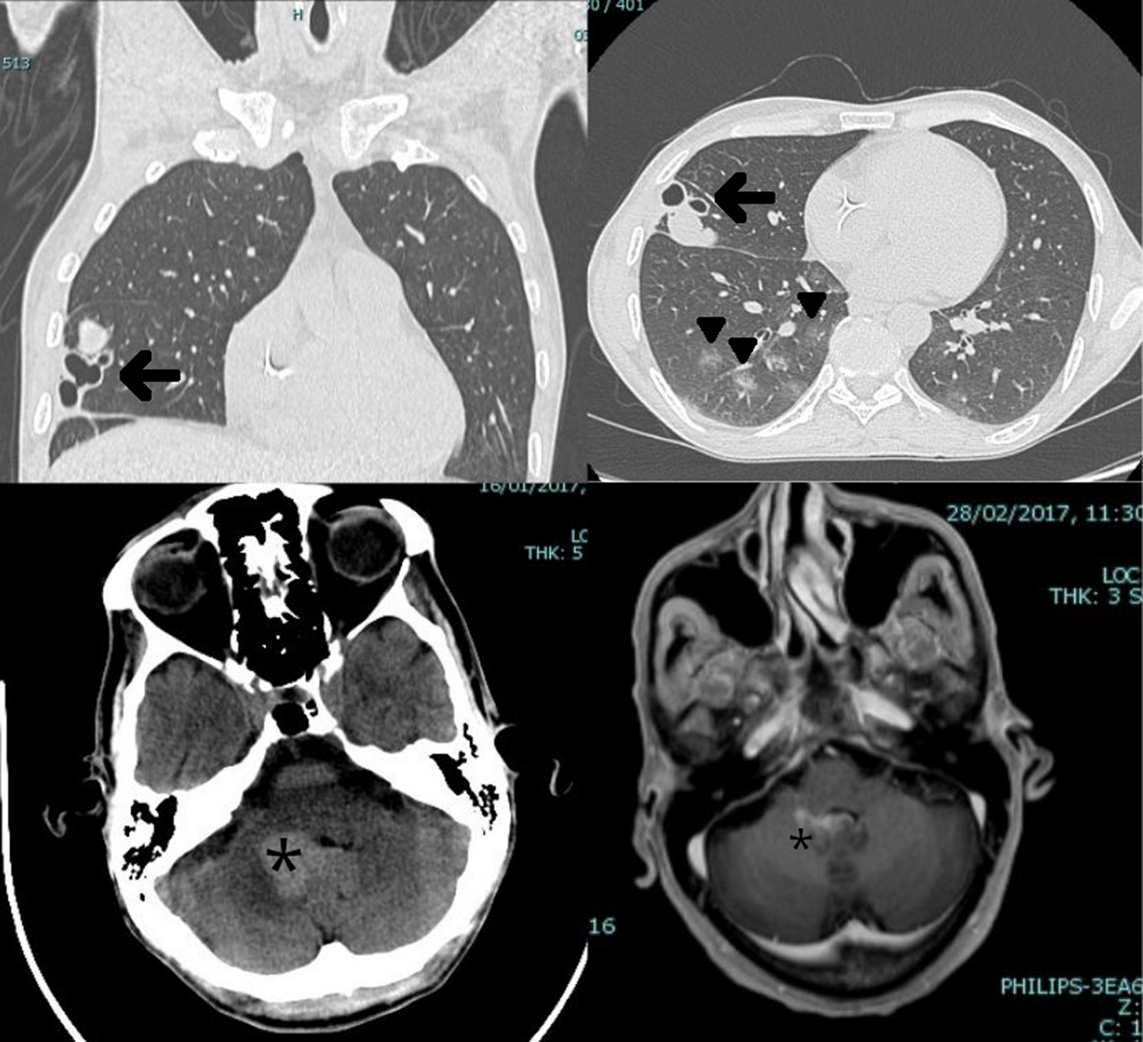
Top: coronal and axial chest CT scans demonstrating thick‐walled cysts (arrows) and ground‐glass opacities (arrowheads). Bottom: brain CT and MR imaging. The cerebellum was involved by an irregular lesion centered on the right tonsil and impinging on the fourth ventricle (asterisk)

A 45‐year‐old North African man presented to the emergency room of our University Hospital complaining of acute headache and generalized weakness and malaise. Chest computed tomography (CT) revealed some centrilobular ill‐defined ground glass opacities, particularly in the mid and upper zones. In the middle lobe, a few thick‐walled irregular cysts were also present (Figure 1). Lung volumes were preserved and no pleural effusions were present. Non‐contrast brain CT demonstrated a large hyperdense area centered on the right cerebellar tonsil causing a slight compression of the fourth ventricle (Figure 1). The clinical differential diagnosis included disseminated tuberculosis, metastatic neoplasm, lymphoma, and Langerhans cell histiocytosis. Further imaging studies with brain and brainstem MRI revealed infiltration of the leptomeninges and ependyma, and perineural involvement of the left trigeminal nerve, and excluded a primary brain tumor.

A fine‐needle aspiration biopsy of one of the pulmonary nodules yielded mostly necrotic material which was inadequate for diagnosis. The Ziehl–Neelsen stain and microbiologic analysis did not support tuberculosis. A spinal tap was performed and the material was cytocentrifuged and Papanicolaou‐stained according to standard protocols.

**FIGURE 2:**
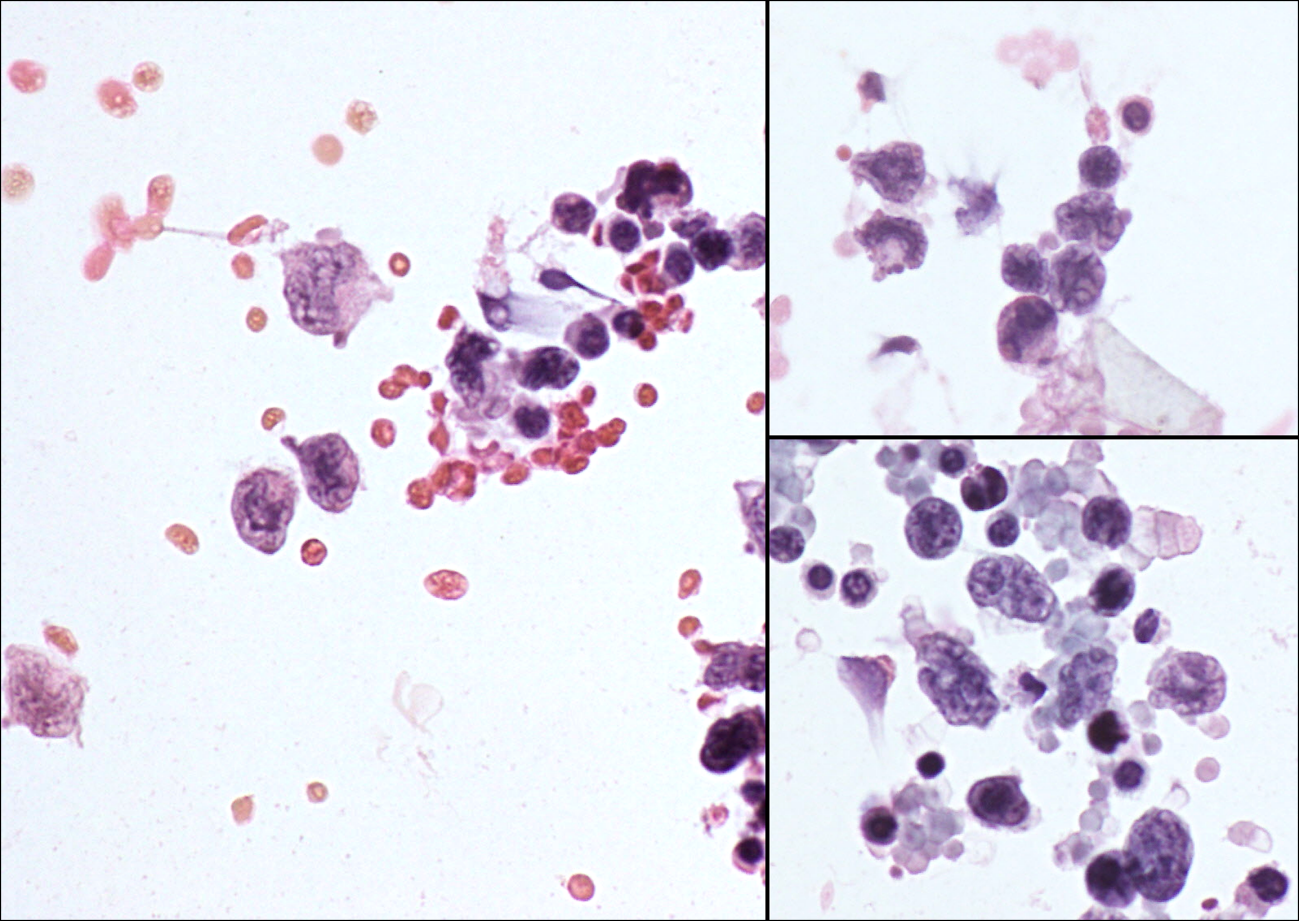
Cytological aspects of Langerhans cell histiocytosis of the cerebrospinal fluid. Numerous large mononuclear cells with cleaved nuclei are set against a background of inflammatory cells and erythrocytes. Inset: detail. (Cytospin, Papanicolaou, ×400)

**FIGURE 3:**
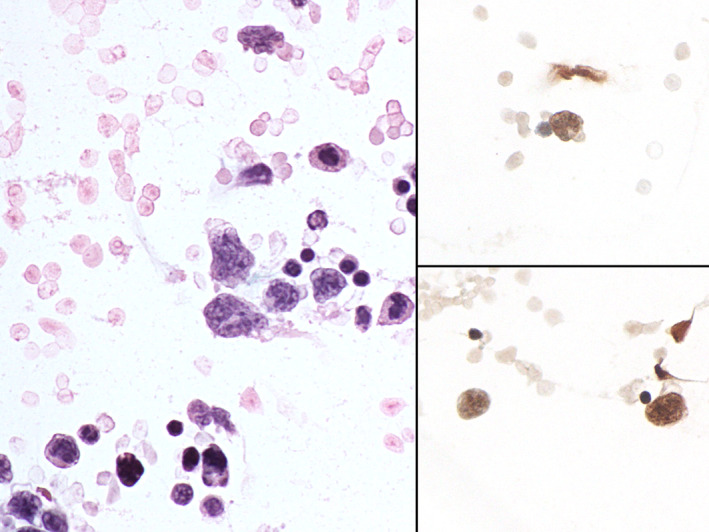
Cytological aspects of Langerhans cell histiocytosis of the cerebrospinal fluid. Some of the large mononuclear cells show degenerative changes. Inset: immunocytochemical positivity for CD1a in the Langerhans cells. (Cytospin, Papanicolaou, ×400; inset: peroxidase‐diaminobenzidine, ×400)

The cytological picture was moderately cellular, with a background of mature lymphocytes, neutrophils and rare eosinophils, in which larger cells with clefted nuclei, consistent with Langerhans cells, could be identified (Figure 2). According to the clinical indications and the cytological features, a basic immunocytochemical panel was performed on the residual material which consisted in only five additional cytospin slides. This revealed positivity for langerin and CD1a (Figure 3). Pan‐cytokeratin (AE1/AE3), CD20, and CD3 were negative in the large cells. A subsequent tru‐cut lung biopsy confirmed the diagnosis.

Meanwhile, the situation deteriorated rapidly with the patient developing an anterolateral myocardial infarction, monolateral vocal cord paralysis, ataxia and dysphagia. Therapy with vinblastine was initiated but unfortunately the patient died only 5 weeks later.

## 3 DISCUSSION

We present the first case of Langerhans cell histiocytosis diagnosed on cytological material from the cerebrospinal fluid.

The cytologic examination of CSF in LCH patients can be normal or show only lymphocytic meningitis, and thus direct CSF involvement by LCH is probably underdiagnosed.^6^, ^7^ The only report of such case in the international literature was published in 2001 by Ghosal and colleagues, who speculate that this unusual localization could be due to involvement of the choroid plexus or to direct invasion of a skull lesion through the dura.^6^

Other serous cavities, such as the pleura, can be involved by LCH. In these few cases, the pleural fluid shows a serofibrinous appearance with high levels of proteins and lactate dehydrogenase, in a background of numerous atypical mononuclear cells. The pleura is thickened and sclerotic, with vascular proliferation and histiocytic infiltration.^8^ The Langerhans cell, in effusions, can be different from its usual appearance: it can show more nuclear irregularities and a rough chromatin pattern, sometimes with giant nucleoli.^3^ The pericardial fluid can also very rarely be involved by LCH.^9^, ^10^

Cytology is a versatile tool which can be used to obtain material both to render a diagnosis and to perform further studies.^11^, ^12^ Fine‐needle aspiration cytology can sample many lesions, both superficial and deep, with good diagnostic performance and minimal costs and invasiveness.^13^, ^14^ The obtained material can be used for special techniques such as immunocytochemistry, flow cytometry, and molecular analyses. Should the material be scanty, the morphologic aspects can be recorded by whole‐slide imaging and then the material can be sacrificed for further tests.^15^, ^16^

Cytological diagnosis of LCH is possible and relies on recognition of the typical cytomorphological features and subsequent immunocytochemical confirmation.^4^, ^17^ Smears will usually be moderately to highly cellular, with Langerhans cells, multinucleated giant cells, lymphocytes, and neutrophils. Abundant eosinophils are seen only in a minority of cases.^17^ Differential diagnosis, mainly when LCH has not yet been diagnosed, may be pointed out with different malignancies, including metastatic neoplasms, primary central nervous system tumors, and high‐grade lymphomas. In our case, because of the shortage of diagnostic material, differential diagnosis depended on the combination of clinical data, radiological features, and cytomorphology. In this way, primary CNS tumors were excluded based on the clinicoradiological features; metastatic carcinoma and NHL could be excluded based on pan‐cytokeratin, CD20, and CD3 negativity. Moreover, cytological features of the neoplastic cells were highly suggestive for LCH and the positivity for CD1a and langerin confirmed this diagnosis.

It is possible that CSF involvement by LCH portends a worse prognosis, however, given the rarity of such finding and the limited data available, no conclusions can be drawn.

In conclusion, we reported the first case of Langerhans cell histiocytosis diagnosed on cerebrospinal fluid. We confirm that LCH can involve the cerebrospinal fluid, and speculate that this may represent an ominous sign. Finally, we confirm that diagnosis of LCH on cytological material can be performed with relative ease by combining morphology and immunocytochemistry.

## AUTHOR CONTRIBUTIONS

Alessandro Caputo conceived the study, drafted the original manuscript, gathered data, performed supervision, and edited the manuscript for important intellectual content. Francesco Tommasino drafted the original manuscript, gathered data, and edited the manuscript for important intellectual content. Chiara Cardamone, Vincenzo Tortora, Francesco Sabbatino, Chiara Di Sarno gathered data and edited the manuscript for important intellectual content.

## FUNDING INFORMATION

The present work received no specific funding.

## CONFLICT OF INTEREST

The authors have no conflict of interest to declare.

## Data Availability

Data sharing not applicable to this article as no datasets were generated or analysed during the current study.
